# Improved methods using the reverse transcriptase polymerase chain reaction to detect tumour cells

**DOI:** 10.1038/sj.bjc.6690155

**Published:** 1999-02

**Authors:** S A Burchill, I J Lewis, P Selby

**Affiliations:** Candlelighter's Children's Cancer Research Laboratory, St James University Hospital, Beckett Street, Leeds, LS9 7TF, UK; ICRF Cancer Medicine Research Unit, St James University Hospital, Beckett Street, Leeds, LS9 7TF, UK; Paediatric Oncology, St James University Hospital, Beckett Street, Leeds, LS9 7TF, UK

**Keywords:** reverse transcriptase polymerase chain reaction, poly-A^+^RNA, tumour cell detection

## Abstract

Reverse transcriptase polymerase chain reaction (RT-PCR) is increasingly used to detect small numbers of circulating tumour cells, though the clinical benefit remains controversial. The largest single contributing factor to the controversy of its value is the different approaches to sample processing. The aim of this study was to compare the sensitivity and reproducibility of RT-PCR for the detection of tumour cells after four commonly used different methods of sample processing. Using RT-PCR, one tumour cell spiked in 2 ml of whole blood was detected after analysis of separated mononuclear cell RNA, whole blood total or poly-A^+^RNA. No false positives were identified with any method. However, the reproducibility of tumour cell detection was reduced after isolation of the mononuclear cell fraction. Only analysis of poly-A^+^RNA had a sensitivity of 100% in all the cell spiking experiments. In patient blood samples, analysis of poly-A^+^RNA increased the number of blood samples positive for tyrosine hydroxylase (TH) mRNA compared with those positive after analysis of total RNA. This may reflect high levels of cDNA reducing the efficiency of the PCR. Isolation of poly-A^+^RNA increases the sensitivity and reproducibility of tumour cell detection in peripheral blood. *© 1999 Cancer Research Campaign*


*British Journal of Cancer* (1999) **79**(5/6), 971–977
© 1999 Cancer Research Campaign
Article no. bjoc.1998.0155

### Improved methods using the reverse transcriptase polymerase chain reaction to detect tumour cells

**SA Burchill[1,2], IJ Lewis[3] and P Selby[2]**

[1]Candlelighter's Children's Cancer Research Laboratory, [2]ICRF Cancer Medicine Research Unit and [3]Paediatric Oncology, St James University Hospital, Beckett Street, Leeds LS9 7TF, UK

**Summary** Reverse transcriptase polymerase chain reaction (RT-PCR) is increasingly used to detect small numbers of circulating tumour cells, though the clinical benefit remains controversial. The largest single contributing factor to the controversy of its value is the different approaches to sample processing. The aim of this study was to compare the sensitivity and reproducibility of RT-PCR for the detection of tumour cells after four commonly used different methods of sample processing. Using RT-PCR, one tumour cell spiked in 2 ml of whole blood was detected after analysis of separated mononuclear cell RNA, whole blood total or poly-A$^+$ RNA. No false positives were identified with any method. However, the reproducibility of tumour cell detection was reduced after isolation of the mononuclear cell fraction. Only analysis of poly-A$^+$ RNA had a sensitivity of 100% in all the cell spiking experiments. In patient blood samples, analysis of poly-A$^+$ RNA increased the number of blood samples positive for tyrosine hydroxylase (TH) mRNA compared with those positive after analysis of total RNA. This may reflect high levels of cDNA reducing the efficiency of the PCR. Isolation of poly-A$^+$ RNA increases the sensitivity and reproducibility of tumour cell detection in peripheral blood.

**Keywords:** reverse transcriptase polymerase chain reaction; poly-A$^+$ RNA; tumour cell detection

Reverse transcriptase polymerase chain reaction (RT-PCR) is increasingly used to detect tumour cells in bone marrow, peripheral blood and peripheral stem cell harvests in a number of different tumour types (Johnson et al, 1995). This method is more sensitive than conventional tumour markers or antibody-based techniques for the detection of small numbers of tumour cells, though its clinical value remains controversial. Most studies report a sensitivity of 1–10 cells detected in $1 \times 10^7$ mononuclear cells or 2 ml of whole blood in cell spiking experiments, but the frequency of tumour cell detection in patient samples remains variable. Although protocols vary in sample processing and RT-PCR method, the consistent sensitivity in spiking experiments suggests in most cases different RT-PCR protocols have been optimized to similar sensitivities. A recent collaborative study by the European Organization for Research and Treatment of Cancer demonstrated the largest single contributing factor to this controversy is sample processing (Keilholz et al, 1998). Some investigators isolate mononuclear cells from whole blood by density gradient centrifugation or concentrate the mononuclear cells by red cell lysis, whereas others extract RNA directly from whole blood. We have evaluated different approaches to sample processing in the context of our studies of neuroblastoma cell detection in peripheral blood.

Neuroblastoma is a common solid tumour of childhood showing a wide range of clinical behaviour, from localized tumours in patients with a good prognosis to highly metastatic aggressive tumours with an unfavourable outcome. Because the presence of disseminating disease is associated with tumour relapse and poor outcome (Rogers et al, 1989; Moss and Sanders, 1990; Moss et al, 1991), the need for early markers in the detection of metastasis are important. Catecholamines are secreted by 98% of neuroblastomas, and therefore the first enzyme in the catecholamine pathway, tyrosine hydroxylase (TH), has been used as a target for RT-PCR detection of neuroblastoma cells in peripheral blood, bone marrow and peripheral stem cell harvests (Burchill et al, 1994*a*, 1994*b*; Miyajima et al, 1995, 1996; Kuroda et al, 1997). The clinical value of this technique in neuroblastoma is difficult to assess, with reported frequency of neuroblastoma cell detection in peripheral blood from stage 4 patients at diagnosis varying from 25% (Kuroda et al, 1997) to 100% (Miyajima et al, 1995).

This study was designed to compare the sensitivity and reproducibility of RT-PCR for the detection of neuroblastoma cells in whole blood using four common methods of blood sample processing. The sensitivity of two of these methods was investigated further in a small cohort of blood samples from untreated patients with advanced stage 4 neuroblastoma.

### MATERIALS AND METHODS

### Cell lines and preparation of cell spikes

The neuroblastoma (IMR-32) cell line purchased from the European Collection of Animal Cell Cultures (PHLS, UK) was used in this study. These cells were grown in Dulbecco's Modified Eagle Medium (DMEM)-RPMI 1640 medium plus 10% fetal calf serum (FCS). Experiments using whole blood into which known numbers of IMR-32 cells were added were used to evaluate the sensitivity, specificity and reproducibility of each process of sample preparation. Four sets of cell spikes were prepared; to 2 ml of whole blood, 0, 1, 10, $10^2$, $10^3$, $10^4$ IMR-32 cells were added. The one and ten cells were added by micromanipulation, greater

*Received 14 April 1998*
*Revised 3 July 1998*
*Accepted 14 July 1998*

*Correspondence to:* SA Burchill, Candlelighter's Children's Cancer Research Laboratory, ICRF Cancer Medicine Research Unit, St James University Hospital, Beckett Street, Leeds LS9 7TF, UK

numbers of cells were added by serial dilution of an IMR-32 cell suspension. Experiments using red cell lysis and isolation of mononuclear fractions were performed four times, and experiments with total or poly-A$^+$ RNA isolated from whole blood were performed five times.

### Clinical samples

Blood (4 ml) was taken into EDTA (20 mM) containing tubes from 15 patients diagnosed with advanced stage 4 neuroblastoma (International Neuroblastoma Staging System; Brodeur et al, 1988). All patients had catecholamine-secreting tumours that expressed TH mRNA (results not shown). Blood samples were divided into two 2-ml aliquots and total RNA extracted using Ultraspec as described below. Blood samples from nine healthy volunteers were included as negative controls. Total RNA from patients or healthy volunteers was divided into two samples. One of these samples was analysed by RT-PCR for TH mRNA in total RNA, and, from the second, poly-A$^+$ RNA was isolated and RT-PCR for TH mRNA performed. Parental consent was given for all children from whom blood was taken.

### Preparation of cell spikes for RT-PCR analysis

Blood samples spiked with IMR-32 cells were processed in four different ways.

### *(A) Red cell lysis*

Red cells were lysed in whole blood cell spikes using a whole blood erythrocyte lysing kit (R&D systems, Minneapolis), according to manufacturer's instructions. Briefly, to 2 ml of whole blood cell spike, 2 ml of 1 × lysing buffer was added and vortexed. This was incubated at room temperature for 10 min. Red cell lysis was visible as darkening of the supernatant. The white cells were pelleted by centrifugation for 5 min at 500 ***g***. The supernatant was removed and precipitated cells washed by resuspending in 2 ml of wash buffer, vortexing and centrifugation for 5 min at 500 ***g***. Cells were resuspended in 1 ml of phosphate-buffered saline (PBS) and added directly to Ultraspec for isolation of total RNA as in (C) below.

### *(B) Isolation of white cell fraction*

The white cell fraction was isolated from whole cell spikes using Lymphoprep (Nycomed Pharma AS, Torshov, Norway). To 2 ml of whole blood cell spikes, 4 ml of PBS was added. This was laid over 3 ml of Lymphoprep and blood separated by centrifugation for 20 min at 600 ***g***. After centrifugation, the mononuclear cells form a distinct band at the Lymphoprep/blood interface. Mononuclear cells were isolated using a fine pastette and this fraction washed twice in PBS (pelleting cells at 500 ***g*** × 5 min in between). Isolated cells were resuspended in 1 ml of PBS and added directly to Ultraspec for isolation of RNA as in (C) below.

### *(C) Total RNA extraction*

Total RNA was extracted from whole blood using Ultraspec (Biogenesis, Bournemouth, UK) as previously described (Burchill et al, 1994*a*). Recovered RNA and its purity were measured by OD at 260 and 280 nm. The quality of isolated RNA was confirmed by separation of RNA (1 µg) in a 1 × TBE agarose gel and RT-PCR analysis for β2 microglobulin using the primer pair, CTCGCGC-TACTCTCTCTTTCT and TGTCGGATTGATGAAACCCAG, as described below.

### *(D) Poly-A$^+$ isolation*

From total RNA isolated as in (C), poly-A$^+$ RNA was isolated using oligo(dT)25 beads (Dynal, Oslo, Norway). Briefly, to total RNA [5 µg in 20 µl of diethyl pyrocarbonate-treated (depc) water], 20 µl of the oligo(dT)25 beads in 2 × binding buffer were added. Oligo(dT)25 beads were washed in 2 × binding buffer (20 mM tris-HCl, pH 7.5; 1.0 M lithium chloride; 2.0 mM EDTA) and resuspended at a concentration of 5.0 mg ml$^{-1}$ in 2 × binding buffer before use. Total RNA plus beads was incubated for 10 min at room temperature. Beads with bound poly-A$^+$ RNA were isolated on a magnetic particle concentrator (MPC; Dynal), and the supernatant removed and discarded. Isolated beads were washed in 100 µl of 2 × binding buffer and resuspended in depc-treated water (20 µl). Poly-A$^+$ RNA was eluted from the beads by heating at 65°C for 5 min. Samples were placed on the MPC and supernatant containing poly-A$^+$ retained.

### Reverse transcriptase polymerase chain reaction (RT-PCR)

Total or poly-A$^+$ RNA was amplified for TH mRNA using 50 cycles of PCR as previously described (Burchill et al, 1994*a*). RT-PCR was performed in a Microflow Omni PCR Workstation (Astec Environmetal Systems, Weston-super-Mare, UK) and products amplified using a DNA Thermal Cycler 480 (Perkin Elmer, NJ, USA). The primer pair used for amplification was ATC ACC TGG TCA CCA AGT TC and GTG GTG TAG ACC TCC TTC CA. For each sample, an RT-negative control (RT enzyme absent) was included. Water negative controls with no total or poly-A$^+$ RNA were also included. RNA extracted from IMR-32 cells was used as a positive control in all experiments. Amplified products were analysed by separation in a 1% agarose 1 × TBE gel with ϕX174 RF DNA molecular weight markers (Gibco, Paisley, UK), stained with ethidium bromide (0.5 µg ml$^{-1}$) and products visualized under UV light on a transilluminator.

The sensitivity of each method for the detection of TH mRNA was calculated as a percentage:

$$\text{sensitivity} = \frac{\text{number of samples positive or negative for TH mRNA}}{\text{number of samples spiked or not spiked with tumour cells}} \times 100$$

### Analysis of reverse transcriptase reaction

Total or poly-A$^+$ RNA extracted from IMR-32 cells was heated in depc water (10 µl) containing RNAase guard (0.5 µl) to 65°C for 5 min, spun at 13 000 ***g*** briefly and placed on ice. To this was added 2 µl of reaction buffer (100 mM tris-HCl, 500 mM potassium chloride, pH 8.3; Perkin Elmer Cetus), 2 mM of dGTP, dTTP and dATP (Pharmacia Biotech, St Albans, UK), 16 mM magnesium chloride (Sigma, Poole, UK), 0.5 µl of random hexamer primers (Gibco), 10 U MMV reverse transcriptase (Pharmacia Biotech), 5 µCi of [α$^{32}$P]dCTP (Amersham International, UK) and depc water to make the final volume 20 µl. Samples were incubated at 37°C for 60 min, and then 80 µl of buffer A (10 mM tris-HCl, pH 7.5, 1 mM EDTA, 150 mM sodium chloride) added. To each sample, 100 µl of phenol/chloroform/isoamyl alcohol (25:24:1; Sigma) was added. The samples were vortexed and briefly spun at

 © Cancer Research Campaign 1999

**A**

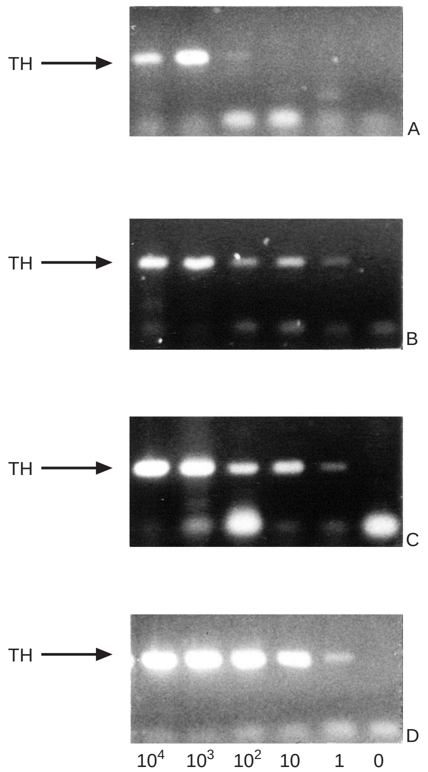

**B**

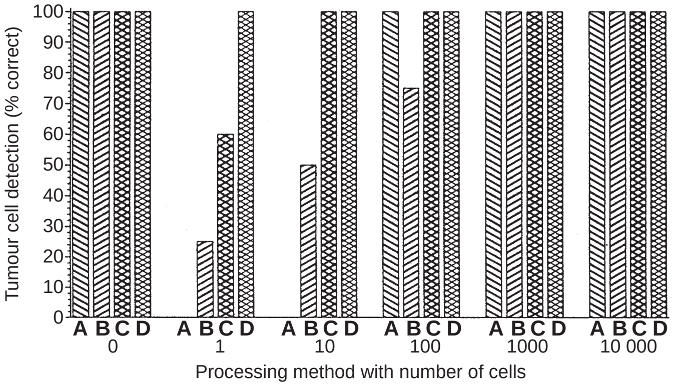

**Figure 1** Sensitivity of RT-PCR for the detection of known numbers of IMR-32 cells in whole blood spikes by RT-PCR for TH mRNA. (**A**) IMR-32 cells (0, 1, 10, $10^2$, $10^3$ or $10^4$) were added to whole blood (2 ml). These spikes were set up four times and analysed for TH mRNA by RT-PCR after processing by: (A) red cell lysis and total RNA extraction; (B) mononuclear cell fractionation and total RNA extraction; (C) whole blood and total RNA extraction; (D) whole blood and poly-$A^+$ RNA extraction. RT-PCR analysis for TH mRNA in total and poly-$A^+$ RNA extracted from IMR-32 cells spiked into whole blood generated a single band of 180 bp, demonstrated on a 1% agarose gel stained with ethidium bromide and visualized under UV light. A single IMR-32 cell was detected in whole blood after procedure B, C, or D. After red cell lysis (procedure A), the sensitivity of IMR-32 cell detection was reduced to 100 cells in 2 ml of whole blood. Primer dimers were visible at the bottom of the gel. (**B**) Percentage of blood samples correct for detection of tumour cells by RT-PCR for TH mRNA. IMR-32 cells (0–$10^4$) were spiked into 2 ml of whole blood and analysed for TH mRNA. The percentage of correct analysis is plotted against IMR-32 cell number added to whole blood

13 000 ***g*** to separate into two phases. The upper aqueous phase was collected, and unincorporated radionucleotide removed by separation through a sepharose Nick column (Sepharose G50, Pharmacia Biotech). Collected fractions (200 µl) were analysed by electrophoresis (40 µl) in an acrylamide gel, which was dried and exposed to film to produce an autoradiograph. Negative controls containing no MMV reverse transcriptase or heat-inactivated RT were included.

### RESULTS

### RT-PCR analysis for TH mRNA in blood samples spiked with IMR-32 cells

RT-PCR for TH mRNA in blood samples spiked with IMR-32 cells generated a single band of 180 bp (Figure 1), as previously described (Burchill et al, 1994*a*). No transcripts were identified in RT-negative or water control samples (results not shown). The

*© Cancer Research Campaign 1999*

**Table 1** Sensitivity and frequency of IMR-32 cell detection in whole blood spikes

| | Number of cells spiked into 2 ml of whole blood | | | | | | Sensitivity (%) |
|---|---|---|---|---|---|---|---|
| | 0 | 1 | 10 | $10^2$ | $10^3$ | $10^4$ | |
| (A) Red cell lysis (*n* = 4) | – | – (0) | – (0) | + (100) | + (100) | + (100) | 60 |
| (B) Mononuclear cell fraction (*n* = 4) | – | + (25) | + (50) | + (75) | + (100) | + (100) | 70 |
| (C) Total RNA (*n* = 5) | – | + (75) | + (100) | + (100) | + (100) | + (100) | 92 |
| (D) Poly-A$^+$ RNA (*n* = 5) | – | + (100) | + (100) | + (100) | + (100) | + (100) | 100 |

Whole blood (2 ml) was spiked with IMR-32 cells (1–$10^4$) and RNA (1 µg) analysed by RT-PCR for TH mRNA after: (A) red cell lysis and total RNA extraction; (B) mononuclear cell fraction and total RNA extraction; (C) whole blood and total RNA extraction; (D) whole blood and poly-A$^+$ RNA extraction. The sensitivity of TH mRNA detection was scored; + = TH mRNA detected, – = TH mRNA not detected. The reliability of these results were assessed in four or five separate experiments (*n*); the sensitivity of detection is given as a percentage in brackets.

**A**

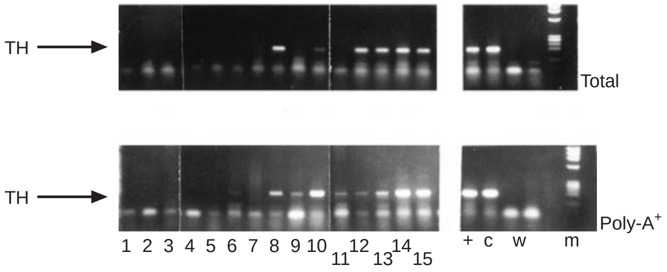

**B**

| | Total RNA | | | Poly-A$^+$ isolated from | | |
|---|---|---|---|---|---|---|
| | 1 µg | 5 µg | 10 µg | 1 µg | 5 µg | 10 µg of total RNA |
| Number of samples positive for TH mRNA out of 15 (% positive) | 6 (40) | 6 (40) | 4 (27) | 6 (40) | 6 (40) | 9 (60) |

**Figure 2** RT-PCR analysis of total RNA or poly-A$^+$ RNA extracted from neuroblastoma patient blood samples. (**A**) Blood samples from 15 patients (1–15) with advanced neuroblastoma were analysed for TH mRNA by RT-PCR of total or poly-A$^+$ RNA. The frequency of TH mRNA detection in total and poly-A$^+$ RNA samples was compared. RNA (100 ng and 10 ng) extracted from IMR-32 cells was included as a positive control (+c), and samples containing no RNA as negative controls (w). The figure shows amplification of 5 µg of total RNA and poly-A$^+$ isolated from 10 µg of total RNA. m = φX174 RF DNA molecular weight marker. (**B**) Summary of TH mRNA detection in total and poly-A$^+$ RNA extracted from 15 blood samples from patients with advanced neuroblastoma identity of the 180-bp band was confirmed to be TH mRNA by Southern blotting and direct sequence analysis. The sensitivity of tumour cell detection by RT-PCR for each of the processing methods was: (A), 60%; (B), 70%; (C), 92%; and (D), 100%; these differences were statistically different using Fisher's exact test ($P < 0.001$). The specificity of TH mRNA detection by RT-PCR in whole blood alone was 100% using any of the isolation procedures (Figure 1B). Amplification for β2 microglobulin generated an RT-PCR product of 136 bp in all isolated RNA or poly-A$^+$ samples (results not shown).

### *(A) Red cell lysis*

In cell spiking experiments, it was possible to detect 100 IMR-32 cells diluted in 2 ml of whole human blood after red cell lysis and extraction of total RNA (Figure 1). In four out of four separate experiments, the level of detection was 100 cells. The sensitivity of detecting TH mRNA in total RNA after red cell lysis was 60% (Table 1).

### *(B) Isolation of white cell fraction*

Using RT-PCR for TH mRNA in RNA extracted from mononuclear cell fractions, it was possible to detect one cell in 2 ml of whole blood (Figure 1). However, this level of sensitivity was only achieved in one out of four experiments, a detection sensitivity of ten cells in 2 ml of whole blood was found in two out of three experiments. In three out of four experiments 100 cells were detected, and in four out of four experiments 1000 cells. The sensitivity of detecting TH mRNA after isolation of the white cell fraction was 70% (Table 1).

### *(C) Total RNA extraction*

TH mRNA was detected by RT-PCR in the one and ten cells spiked into 2 ml of whole blood (Figure 1). TH mRNA was detected in three out of five of the one cell and five out of five of the ten cells in 2 ml of whole blood spikes. The sensitivity of detecting TH mRNA in total RNA extracted from whole blood was 92%.

 *© Cancer Research Campaign 1999*

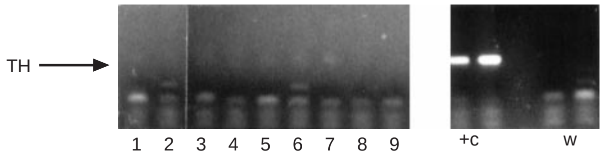

**Figure 3** RT-PCR analysis of poly-A+ RNA extracted from normal control blood samples. Poly-A+ RNA was extracted from normal control blood (2 ml) from nine age-matched children (1–9). RNA (100 ng and 10 ng) extracted from IMR-32 cells was included as a positive control (+c), and samples containing no RNA as negative controls (w)

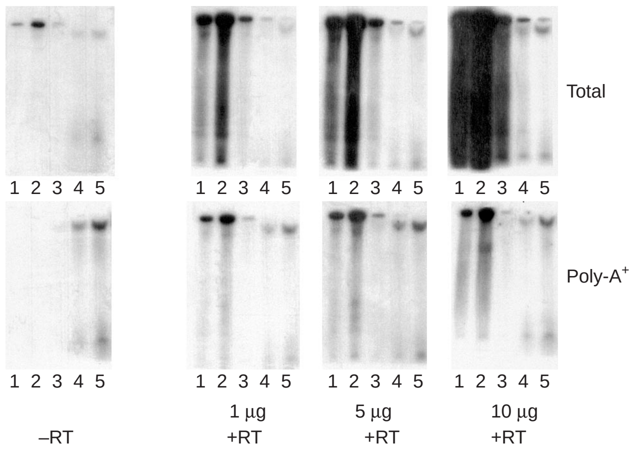

**Figure 4** cDNA synthesis from isolated total or poly-A+ RNA was assessed by measuring incorporation of [$\alpha^{32}$P]dCTP. Total or poly-A+ mRNA (1, 5, 10 µg) was transcribed to cDNA as in the RT-PCR reaction, except dCTP was replaced with radiolabelled [$\alpha^{32}$P]dCTP. Radiolabelled cDNA, separated from unincorporated radioactivity using a Nick column, was found in fractions 1, 2 and 3 (after the 400-µl dead volume). Each fraction (1–5) is a volume of 200 µl. No cDNA was detected when total or poly-A+ RNA from 10 µg of RNA was incubated in the absence of MMV reverse transcriptase (–RT)

*(D) Poly-A+ isolation*

After amplification of poly-A+ RNA for TH mRNA, it was possible to detect one cell in 2 ml of whole blood (Figure 1). The detection sensitivity after isolation of poly-A+ RNA from whole blood was 100%, TH mRNA was detected in five out of five experiments at the level of one cell in 2 ml of whole blood (Table 1).

### RT-PCR analysis of clinical samples

Analysing 1 µg of total RNA extracted from peripheral blood of patients with stage 4 neuroblastoma, 6 out of 15 (40%) were positive for TH mRNA: patient samples 8, 10, 12, 13, 14 and 15 (Figure 2; Table 2). Identical results were obtained when 5 µg of total RNA was analysed. However, when larger amounts of RNA were analysed (10 µg), the frequency of TH mRNA detection was reduced to 4 out of 15 (27%) (Figure 2B). Analysis of poly-A+ RNA isolated from matched blood samples gave the same results when 1 and 5 µg of RNA were analysed, i.e. 6 out of 15 blood samples were positive for TH mRNA (40%). The same samples were positive after analysis of total and poly-A+ RNA. However, when 10 µg of RNA were analysed, 9 out of 15 (60%) of samples were positive for TH mRNA (Figure 2): the six blood samples previously positive for TH mRNA after analysis of 1 and 5 µg of RNA (patient samples 8, 10, 12, 13, 14, 15) and an additional three (6, 9, 11). No TH mRNA was detected in total or poly-A+ RNA extracted from the nine control normal blood samples (Figure 3). All total RNA or poly-A+ RNA samples were positive for β2 microglobulin (results not shown).

### Analysis of reverse transcriptase reaction

Radiolabelled cDNA was eluted from the Nick column in fractions 1, 2 and 3, after the column dead volume (400 µl) (Figure 4). The peak of cDNA was recovered in fraction 2. Incubation of total or poly-A+ RNA with heat-inactivated or no MMV reverse transcriptase did not produce cDNA (Figure 4). The amount of cDNA produced was proportional to the amount of total or poly-A+ RNA added, but was lower in the poly-A+ than the total RNA samples.

### DISCUSSION

Using RT-PCR for TH mRNA, it was possible to detect a single IMR-32 neuroblastoma cell in 2 ml of whole blood, consistent with previously published data (Burchill et al, 1994*a*,*b*). However, the sensitivity and specificity of tumour cell detection was dependent on the method of sample processing. No false positives were detected in the cell spiking experiments, i.e. TH mRNA was not detected in any of the unspiked blood samples. However, analysis for TH mRNA after red cell lysis or mononuclear cell isolation led to eight and six false negatives respectively. The high number of false negatives suggests loss of tumour cells or isolation of poor quality RNA using these two techniques. Loss of tumour cells from the isolated mononuclear cell fraction using lymphoprep would most likely explain the decreased reproducibility in procedure B. Previous studies have demonstrated loss of melanoma cells after density gradient separated mononuclear cell fractions (Keilholz, 1996). Degradation of mRNA can be a particularly difficult problem when analysing circulating tumour cells in blood, bone marrow or peripheral blood stem cells (PBSCs) because red blood cells contain high levels of RNAases, which once released will rapidly degrade mRNA (Jackson et al, 1991). Red cell lysis may have lysed a proportion of tumour cells, which may then be exposed to RNAases released from the red blood cells. Equally, contamination of isolated RNA with a substance which reduces the efficiency of the PCR amplification may also contribute to the false negatives, e.g. prophyrin compounds derived from haem can inhibit DNA polymerase activity (Higuchi, 1989).

The sensitivity of TH mRNA detection in whole blood spiked samples by RT-PCR was 92% and 100%, after analysis of total RNA or poly-A+ RNA respectively. This increase in sensitivity, when compared with analysis of total RNA after red cell lysis (60%) or mononuclear cell fractionation (70%), demonstrates analysis of whole blood is more reliable, probably by avoiding loss of tumour cells. The number of tumour cells lost after either red cell lysis or mononuclear cell fractionation will vary depending on susceptibility to lysis or size of different tumour cell populations. This heterogeneity of tumour cells within and between cancers strengthens the case for analysis of whole blood. Although RNA isolated from whole blood is often contaminated with DNA, providing primers for the target to be amplified are selected in exons separated by an intron, theoretically this should have no effect on the amplification efficiency. However, large amounts of contaminating genomic DNA are reported to reduce the efficiency of PCR amplification despite the design of primers in different exons (M Willhauk, personal communication). In this study, increasing the amount of total RNA in an RT-PCR can result in loss of sensitivity. This inhibition of PCR

© Cancer Research Campaign 1999

efficiency limits the amount of RNA and consequently blood volume that may be analysed in any single PCR. Amplification of poly-A+ RNA isolated from whole blood total RNA was more sensitive than analysis of total RNA alone. Because poly-A+ RNA comprises less than 5% of the total RNA within a cell, isolation of poly-A+ RNA before reverse transcriptase would reduce the amount of cDNA produced from tRNA and rRNA, and also reduce the level of contaminating DNA. This allows analysis of larger amounts of RNA and therefore blood volumes, leading to an increase in sensitivity of tumour cell detection. Previous studies have suggested analysis of whole blood RNA may be less sensitive than analysis of RNA after red cell lysis (Gläser et al, 1997) or ficol separation (Gläser et al, 1997; Jung et al, 1997). This study demonstrates isolation of poly-A+ from total RNA will increase the sensitivity of whole blood analysis. The relationship between detection of TH mRNA in patient samples after analysis of poly-A+ RNA rather than total RNA was not linear; the frequency of TH mRNA detection in patient samples was the same when 1 or 5 μg of total or poly-A+ RNA was analysed. The discrepancy between cell spiking experiments, in which a linear relationship was found, and patient sample analysis may reflect variations in the level of TH mRNA in cell line and patient sample RNA. The heterogeneity of TH mRNA expression is currently under investigation in a wider cohort of cell lines and primary tumours; neuroblastomas that do not express TH mRNA would not be detected by this method. It is unlikely that poly-A+ RNA isolated from patient samples would be contaminated with DNA or a factor which is reducing the efficiency of the amplification any more than in the cell spikes.

The patient blood samples analysed in this study were taken at diagnosis from patients with stage 4 disease, before treatment. This group of patients was selected as they had clinically proven, untreated stage 4 disease, and would therefore be most likely to have circulating tumour cells. However, circulating tumour cells were only detected in 60% of stage 4 patients using the most sensitive method of sample processing, poly-A+ isolation. Failure to detect circulating tumour cells in all stage 4 blood samples probably reflects the random shedding of tumour cells into the circulation (Fidler, 1990). Preliminary studies suggest the detection of circulating tumour cells in stage 4 neuroblastoma patients at diagnosis may identify a subgroup of patients with a worse prognosis than those patients in which tumour cells are not detected (Burchill et al, 1994*b*; Miyajima et al, 1995; Kuroda et al, 1997), though the number of patients in these studies are small. The clinical significance of tumour cell detection by RT-PCR for TH mRNA is currently being investigated nationally through the United Kingdom Children's Cancer Study Group (study number NB 9305).

Although RT-PCR detection of circulating tumour cells has been shown to increase the sensitivity of small-volume disease detection, the clinical significance of detecting low levels of disease remains unclear, and transfer of the technology into a clinical setting has been slow. This reflects the lack of good quality, long-term clinical outcome studies and the technical challenges associated with RNA-based assays. These studies demonstrate collection of whole blood samples into EDTA, rapidly frozen at –80°C, and subsequent extraction of poly-A+ RNA is a suitable and reliable method for the processing of blood samples before analysis by RT-PCR. Thus, sample collection in the clinic can be as uncomplicated as taking a whole blood sample into EDTA and freezing at –80°C, this will ease sample collection for the much needed multicentre quality controlled studies to evaluate the clinical significance of this technique in small-volume disease detection.

In summary, these studies support the hypothesis that sample preparation, RNA extraction and cDNA synthesis account for most of the heterogeneity of RT-PCR assay results in patient samples. No false negatives or positives were detected after RT-PCR analysis of poly-A+ RNA isolated from whole blood cell spikes, and an increased frequency of tumour cell detection was found in patient blood samples. Further studies are required to investigate the relationship between tumour cell shedding, frequency of blood sampling and the blood volume analysed, which are important biological variables. Molecular staging of human cancers by evaluating the primary tumour and the circulation of potentially metastatic cells is a vital and attainable goal for clinical cancer research. Existing procedures are an important beginning for evaluation of the clinical significance of such methods, but progress can only be made if we rigorously evaluate them scientifically and technically.

### ACKNOWLEDGEMENTS

This work has been funded by the Candlelighter's Trust, the Neuroblastoma Society and the Northern and Yorkshire Regional Health Authority. Thank you to Mr FM Bradbury and Mrs R Harrison for technical assistance, and to Mrs M Jones for statistical advice.

### REFERENCES

Brodeur GM, Seeger RC, Barrett A, Berthold F, Castleberry RP, D'Angio G, De Bernardi B, Evans AE, Favrot M, Freeman AI, Haase G, Hartmann O, Hayes FA, Hebon L, Kemshead J, Lampert F, Ninane J, Ohkowa H, Philip T, Pinkerton CR, Pritchard J, Sawada T, Siegel S, Smith EI, Tsuchida Y and Voute PA (1988) International criteria for diagnosis, staging and response to treatment in patients with neuroblastoma. *J Clin Oncol* **6**: 1874–1881

Burchill SA, Bradbury FM, Smith B, Lewis IJ and Selby P (1994*a*) Neuroblastoma cell detection by reverse transcriptase-polymerase chain reaction (RT-PCR) for tyrosine hydroxylase mRNA. *Int J Cancer* **57**: 671–675

Burchill SA, Bradbury FM and Lewis IJ (1994*b*) Early clinical evaluation of reverse transcriptase-polymerase chain reaction (RT-PCR) for tyrosine hydroxylase. *Eur J Cancer* **31A**: 553–556

Fidler I (1990) Critical factors in the biology of human cancer metastasis. *Cancer Res* **50**: 6130–6138

Gläser R, Rass K, Seiter S, Hauschild A, Christophers E and Tilgen T (1997) Detection of circulating melanoma cells by specific amplification of tyrosinase complementary DNA is not a reliable tumour marker in melanoma patients: a clinical two centre study. *J Clin Oncol* **15**: 2818–2825

Higuchi R (1989) Principles and applications for DNA amplification. In *PCR Technology.* Erlich H (ed.), pp. 31–43. Stockton Press, New York, USA

Jackson DP, Hayden JD and Quirke P (1991) *PCR. A Practical Approach.* McPherson MJ, Quirke P and Taylor GR (eds.), pp. 29–50. IRL Press

Johnson P, Burchill SA and Selby P (1995) The molecular detection of tumour cells. *Br J Cancer* **72**: 268–276

Jung FA, Buzaid AC, Ross MI, Woods KV, Lee JJ, Albitar M and Grimm EA (1997) Evaluation of tyrosinase mRNA as a tumour marker in the blood of melanoma patients. *J Clin Oncol* **15**: 2826–2831

Keilholz U (for the EORTC Melanoma Cooperative Group) (1996) Diagnostic PCR in melanoma; methods and quality assurance (review). *Eur J Cancer* **32**: 1661–1663

Keilholz U, Willhauck M, Rimoldi D, Brasseur F, Dummer W, Rass K, De Vries T, Blaheta J, Voit C, Lethe B and Burchill SA (for EORTC-MCG) (1998) Reliability of RT-PCR based assays for detection of circulating tumour cells: a quality-assurance initiative of the EORTC Melanoma Cooperative Group. *Eur J Cancer* **34**: 750–753

Kuroda T, Saeki M, Nakano M and Mizutani S (1997) Clinical application of minimal residual neuroblastoma cell detection by reverse transcriptase-polymerase chain reaction. *J Pediatr Surg* **32**: 69–72

Miyajima Y, Kato K, Numata S, Kudo K and Horibe K (1995) Detection of neuroblastoma cells in bone marrow by the reverse transcriptase polymerase chain reaction for tyrosine hydroxylase mRNA. *Cancer* **75**: 2757–2761

 © Cancer Research Campaign 1999

Miyajima Y, Horibe K, Fukuda M, Matsumoto K, Numata S, Mori H and Kato K (1996) Sequential detection of tumor cells in the peripheral blood and bone marrow of patients with stage IV neuroblastoma by the reverse transcription polymerase chain reaction for tyrosine hydroxylase mRNA. *Cancer* **77**: 1214–1219

Moss TJ and Sanders DG (1990) Detection of neuroblastoma cells in blood. *J Clin Oncol* **8**: 736–740

Moss TJ, Reynolds CP and Sather HN (1991) Prognostic value of immunocytological detection of bone marrow metastases in neuroblastoma. *N Engl J Med* **324**: 219–226

Rogers DW, Treleaven JG, Kemshead JT and Pritchard J (1989) Monoclonal antibodies for detecting bone marrow invasion by neuroblastoma. *J Clin Pathol* **42**: 422–426

*© Cancer Research Campaign 1999*

## References

[PDF_00512] Brodeur G. M., Seeger R. C., Barrett A., Berthold F., Castleberry R. P., D'Angio G., De Bernardi B., Evans A. E., Favrot M., Freeman A. I. (1988). International criteria for diagnosis, staging, and response to treatment in patients with neuroblastoma.. J Clin Oncol.

[PDF_00521] Burchill S. A., Bradbury F. M., Selby P., Lewis I. J. (1995). Early clinical evaluation of neuroblastoma cell detection by reverse transcriptase-polymerase chain reaction (RT-PCR) for tyrosine hydroxylase mRNA.. Eur J Cancer.

[PDF_00518] Burchill S. A., Bradbury F. M., Smith B., Lewis I. J., Selby P. (1994). Neuroblastoma cell detection by reverse transcriptase-polymerase chain reaction (RT-PCR) for tyrosine hydroxylase mRNA.. Int J Cancer.

[PDF_00524] Fidler I. J. (1990). Critical factors in the biology of human cancer metastasis: twenty-eighth G.H.A. Clowes memorial award lecture.. Cancer Res.

[PDF_00526] Gläser R., Rass K., Seiter S., Hauschild A., Christophers E., Tilgen W. (1997). Detection of circulating melanoma cells by specific amplification of tyrosinase complementary DNA is not a reliable tumor marker in melanoma patients: a clinical two-center study.. J Clin Oncol.

[PDF_00534] Johnson P. W., Burchill S. A., Selby P. J. (1995). The molecular detection of circulating tumour cells.. Br J Cancer.

[PDF_00536] Jung F. A., Buzaid A. C., Ross M. I., Woods K. V., Lee J. J., Albitar M., Grimm E. A. (1997). Evaluation of tyrosinase mRNA as a tumor marker in the blood of melanoma patients.. J Clin Oncol.

[PDF_00539] Keilholz U., Willhauck M., Rimoldi D., Brasseur F., Dummer W., Rass K., de Vries T., Blaheta J., Voit C., Lethé B. (1998). Reliability of reverse transcription-polymerase chain reaction (RT-PCR)-based assays for the detection of circulating tumour cells: a quality-assurance initiative of the EORTC Melanoma Cooperative Group.. Eur J Cancer.

[PDF_00547] Kuroda T., Saeki M., Nakano M., Mizutani S. (1997). Clinical application of minimal residual neuroblastoma cell detection by reverse transcriptase-polymerase chain reaction.. J Pediatr Surg.

[PDF_00555] Miyajima Y., Horibe K., Fukuda M., Matsumoto K., Numata S., Mori H., Kato K. (1996). Sequential detection of tumor cells in the peripheral blood and bone marrow of patients with stage IV neuroblastoma by the reverse transcription-polymerase chain reaction for tyrosine hydroxylase mRNA.. Cancer.

[PDF_00550] Miyajima Y., Kato K., Numata S., Kudo K., Horibe K. (1995). Detection of neuroblastoma cells in bone marrow and peripheral blood at diagnosis by the reverse transcriptase-polymerase chain reaction for tyrosine hydroxylase mRNA.. Cancer.

[PDF_00562] Moss T. J., Reynolds C. P., Sather H. N., Romansky S. G., Hammond G. D., Seeger R. C. (1991). Prognostic value of immunocytologic detection of bone marrow metastases in neuroblastoma.. N Engl J Med.

[PDF_00560] Moss T. J., Sanders D. G. (1990). Detection of neuroblastoma cells in blood.. J Clin Oncol.

[PDF_00565] Rogers D. W., Treleaven J. G., Kemshead J. T., Pritchard J. (1989). Monoclonal antibodies for detecting bone marrow invasion by neuroblastoma.. J Clin Pathol.

